# Case Series: Efficacy of Polyclonal Intravenous Immunoglobulin for Refractory *Clostridioides difficile* Infection

**DOI:** 10.3390/antib13020026

**Published:** 2024-04-01

**Authors:** Sophie A. Ragan, Caitlin Doyle, Neha Datta, Heather Abdic, Mark H. Wilcox, Ros Montgomery, Shanika A. Crusz, Yashwant R. Mahida, Tanya M. Monaghan

**Affiliations:** 1 Department of Gastroenterology, Nottingham University Hospitals NHS Trust, Nottingham NG7 2UH, UK; sophie.ragan@nhs.net (S.A.R.); heather.abdic@nuh.nhs.uk (H.A.); 2 School of Medicine, University of Nottingham, Nottingham NG7 2RD, UK; caitlin.doyle@nottingham.ac.uk (C.D.); neha.datta@nottingham.ac.uk (N.D.); 3 Healthcare Associated Infection Research Group, Leeds Institute of Medical Research, University of Leeds, Leeds LS9 7TF, UK; mark.wilcox@nhs.net; 4 Department of Microbiology, Leeds Teaching Hospitals, Leeds LS1 3EX, UK; 5 Infection and Prevention Control, Nottingham University Hospitals NHS Trust, Nottingham NG7 2UH, UK; ros.montgomery@nuh.nhs.uk; 6 Department of Microbiology, Nottingham University Hospitals NHS Trust, Nottingham NG7 2UH, UK; shanika.crusz@nuh.nhs.uk; 7 NIHR Nottingham Biomedical Research Centre, School of Medicine, University of Nottingham, Nottingham NG7 2UH, UK; yash.mahida@nottingham.ac.uk; 8 Nottingham Digestive Diseases Centre, University of Nottingham, Nottingham NG7 2UH, UK; 9 Translational Medical Sciences, School of Medicine, University of Nottingham, Nottingham NG7 2UH, UK

**Keywords:** intravenous immunoglobulin, *Clostridioides difficile* infection, case series

## Abstract

Background: Intravenous immunoglobulin (IVIg) for *Clostridioides difficile* infection (CDI) no longer features in treatment guidelines. However, IVIg is still used by some clinicians for severe or recurrent CDI (rCDI) cases. The main objective of this study was to investigate the efficacy of IVIg and to identify possible predictors of disease resolution post IVIg administration for patients with CDI. Methods: This retrospective observational cohort study of patients ≥2 years old hospitalised with severe, relapsing, or rCDI treated with IVIg therapy was performed in a large UK tertiary hospital between April 2018 and March 2023. Scanned electronic notes from patient admissions and clinical reporting systems were used to collect relevant data. Results: In total, 20/978 patients diagnosed with CDI over the 5-year study were treated with IVIg. Twelve (60%) had hospital-onset CDI. Eleven of the twenty patients (55%) responded to treatment, with a mean of 8.6 (SD 10.7) days to disease resolution. Sixteen (80%) patients were treated for severe CDI and four (20%) for rCDI (n = 3) and relapsing CDI (n = 1). There were no statistically significant differences in possible independent predictors of disease resolution post IVIg administration between groups. There was an average of 6.2 (4.9) days to IVIg administration after diagnosis with no difference between responders and non-responders (*p* = 0.88) and no further significant difference in additional indicators. Four (36%) of the responders were immunosuppressed compared to just one (11%) of the non-responders (*p* = 0.15). Six of the responders (two with recurrent and four with severe CDI) improved rapidly within 2 days, and three of these were immunosuppressed. Conclusion: We observed disease resolution post IVIg therapy in over 50% of patients with refractory CDI. Our data also support a potential enhanced effect of IVIg in immunosuppressed individuals. Thus, the role of IVIg for CDI treatment, particularly in the immunosuppressed, warrants future case–control studies coupled to mechanistic investigations to improve care for this ongoing significant healthcare-associated infection.

## 1. Introduction

*Clostridioides difficile* infection (CDI) constitutes a critical public health challenge globally [1]. The worldwide reported incidence rate of CDI ranges from 1.1 to 631.8 per 100,000 population per year [2], probably majorly affected by ascertainment biases. However, it remains challenging to accurately estimate the true healthcare and economic burden of CDI given the lack of a concerted global effort for epidemiological surveillance, particularly in low- and middle-income countries [3]. CDI is the leading cause of healthcare-associated infective diarrhoea and is increasingly being linked to community-acquired cases of colitis [4,5]. Severe CDI remains a substantial treatment challenge and unmet therapeutic need. It is defined as CDI not responding to recommended CDI treatment and can be part of either non-complicated or severe-complicated CDI, the latter of which is defined by the presence of hypotension, septic shock, elevated serum lactate, ileus, toxic megacolon, bowel perforation, or any fulminant course of disease [6]. Recurrent infections refractory to standard treatments are also particularly problematic because they are associated with worse quality of life, longer hospital stays, and higher mortality than index infections [7,8,9].

Treatment strategies for CDI are evolving and are currently determined by risk of recurrence rather than disease severity in the European Society of Clinical Microbiology and Infectious Diseases (ESCMID) guidelines [6]. In patients with an initial CDI, treatment includes standard-of-care antibiotics (SoC) (fidaxomicin (first line) or vancomycin (second line)). In patients at high risk of recurrence, first- and second-line recommended treatments are fidaxomicin and SoC and bezlotoxumab (human monoclonal antibody that binds to and neutralises *C. difficile* toxin B), respectively, or oral metronidazole if the latter options are unavailable. For a first CDI recurrence, SoC and bezlotoxumab or fidaxomicin, or tapering and pulsed vancomycin are recommended. For more than two recurrences, ESCMID recommends Faecal Microbial Transplantation (FMT) as a first-line treatment, SoC and bezlotoxumab as second-line treatments, and finally tapered and pulsed vancomycin if the preferred option is unavailable [6]. For severe CDI, recommended treatments are vancomycin or fidaxomicin +/− adjunctive intravenous metronidazole or tigegycline. These same treatments can be used for severe–complicated and refractory severe CDI together with intravenous tigecycline and FMT in refractory cases with early surgical consultation. The updated Infectious Diseases Society of America (IDSA) and Society for Healthcare Epidemiology of North America (SHEA) recommend similar stratified treatment approaches [10]. Whilst all three societal guidelines recommend bezlotoxumab for recurrent CDI (rCDI), the National Institute of Clinical Excellence (NICE) does not recommend bezlotoxumab due to its high acquisition costs and perceived reduced cost effectiveness compared with SoC treatments, which have recently been questioned [11,12]. NICE recommends vancomycin as a first-line treatment for a first episode of CDI with fidaxomicin as a second-line option. For further episodes of CDI, NICE recommends fidaxomicin for relapsing CDI (within 12 weeks of symptom resolution) and either vancomycin or fidaxomicin for rCDI (a further episode of CDI occurring more than 12 weeks after symptom resolution). For life-threatening CDI, NICE recommends high-dose oral vancomycin with intravenous metronidazole. However, intravenous immunoglobulin (IVIg), another passive immunotherapeutic approach for the treatment of CDI, no longer features in international or societal guidelines. Nonetheless, IVIg is still being used by some clinicians for the treatment of recurrent or severe CDI.

IVIg represents a heterogeneous mixture of immunoglobulins, most abundantly IgG, derived from the plasma of numerous healthy donors [13]. Commercial IVIg preparations contain a large repertoire of antibody specificities, resulting in the neutralisation of a wide range of antigens including pathogens and super antigens [14]. When applied as a treatment for refractory CDI, the multimodal action of IVIg makes it a viable therapeutic candidate. Firstly, the diverse pool of antibodies within IVIg, specifically IgG anti-toxin A and B antibodies, targets *C. difficile* toxins TcdA and TcdB, thereby limiting the disruption of the intestinal epithelial barrier and dampening mucosal inflammation [13,14,15]. Additionally, IVIg modulates the activation and effector functions of B and T lymphocytes, neutralises pathogenic autoantibodies, interferes with antigen presentation, and has a strong anti-inflammatory effect which depends on its interaction with the complement system, cytokines, and endothelial cells [16]. This multifaceted mechanism of action underscores the efficacy of polyclonal IVIg in addressing refractory CDI by both neutralising toxins and modulating the immune response to restore intestinal immune homeostasis [17].

The current evidence for IVIg in CDI is limited with some successful outcomes being reported from small case series and case reports [13,18,19,20,21,22,23,24,25,26,27]. As a result, IVIg is not currently indicated in international societal or UK national guidelines [6]. Variability among FDA-approved IVIg products and uncertainty about their specific efficacy against *C. difficile* toxins further highlight the need for research [21]. Despite this, IVIg is still used by some clinicians to treat severe or recurrent CDI cases. The principal aim of this retrospective study was to investigate the efficacy of IVIg in a small patient cohort in a single UK centre before and after the COVID-19 pandemic in order to help define its use and clinical impact.

## 2. Materials and Methods

### 2.1. Data Source and Study Population

We conducted a single-centre retrospective observational cohort study in Nottingham University Hospitals NHS Trust (NUHT), UK, from April 2018 to March 2023. The cohort comprised both children (≥2 years of age) and adult patients (≥18 years of age) who were hospitalised with severe or rCDI and were treated with IVIg therapy. A diagnosis of CDI was made in patients with new-onset diarrhoea and CDI was confirmed by means of a three-step testing strategy including an initial RIDA^®^ QUICK *C. difficile* glutamate dehydrogenase (GDH) screening test followed by RIDA^®^ QUICK *C. difficile* Toxin A/B toxin enzyme-linked immunoassays (ELISAs). Patient samples testing GDH- and toxin-positive were deemed to have active CDI. In instances of GDH positivity and toxin negativity, a further polymerase chain reaction (PCR) was performed, and treatment was commenced with vancomycin or fidaxomicin in some symptomatic patients with PCR-positive test results with deemed false negative toxin results. Severe disease was defined as a white cell count (WCC) greater than 15 × 10^9^/L, or an acutely increased serum creatinine concentration (greater than 50% above baseline), or a temperature higher than 38.5 °C, or evidence of severe colitis (abdominal or radiological computed tomography signs). Recurrent disease was defined as CDI recurrence occurring more than 12 weeks after previous symptom resolution, whereas relapsing CDI was defined as CDI occurring within 12 weeks of previous infection in accordance with NICE guidance [11]. Community-onset CDI was defined as CDI detected within the first 2 days of admission (where date of admission is day 1), and hospital-onset CDI as CDI detected after the first 2 days of admission [28]. *C. difficile* stool samples were also sent to the Leeds Ribotyping Network Service for ribotyping in instances of severe CDI, cases with environmental links, or where CDI was recorded as part 1 of the death certificate. All patients with rCDI that were identified as suitable for IVIg treatment were discussed with the IVIg approval panel at NUHT. IVIg is approved within NUHT in the following situations: (i) for the treatment of severe CDI where the patient has failed to respond to maximal therapy, vancomycin PO 500 mg Q6H + IV metronidazole 500 mg Q8H (adults), and where maximal therapy has been prescribed for children, especially in patients where surgery is not an option; (ii) to prevent the recurrence of CDI in patients where other therapies (e.g., tapering vancomycin) have failed or are inappropriate. All cases were cross-referenced with the *C. difficile* specialist nurse spreadsheet, digital health records (DHR), patient results and correspondence, drug history and infection prevention, and control team weekly clinical meeting notes. Data were collected across a variety of demographic, clinical, and laboratory domains. Scanned electronic notes from patient admissions and clinical reporting systems were used to collect relevant data. The research was reviewed by the clinical governance team at NUHT, and informed consent was not required since this was a service evaluation and minimal-risk retrospective study using anonymized data.

### 2.2. Exposure and Study Outcomes

Human IVIg preparations including Privigen^®^, Panzyga^®^, Kiovig^®^, and Intratect^®^ 10% were administered (0.4 g/kg), and doses were calculated based on patient weight. The primary objectives were to investigate the efficacy of IVIg for CDI and to identify possible independent predictors of disease resolution post IVIg administration in those with (i) severe and (ii) relapsing and rCDI. Response was assessed as the resolution of symptoms in those with ongoing diarrhoea. The variables examined were age, body mass index (BMI), gender, IVIg indication, Charlson Comorbidity Index, immunosuppression status (immunosuppression defined as use of an immunosuppressant medication, biologic, steroid dose, ≥2 weeks of daily prednisolone of 20 mg/d or 2 mg/kg or equivalent, chemotherapy, or current malignancy), number of medications on admission, prior PPI or recent antibiotics, whether hospital- or community-onset infection, peak WCC, peak C-reactive protein (CRP), lowest albumin (during admission and following CDI diagnosis), and radiological findings [29].

The secondary objectives were to assess the impact of time from symptom onset and diagnosis to the request and administration of IVIg on clinical response and additionally investigate the effects of dosing, demographic, and clinical indicators on IVIg response. These included time to symptom resolution, length of hospital stay, recurrence, surgical intervention, and 30-day mortality between responders and non-responders. Cases where death occurred with an inpatient were excluded from the analysis of length of hospital stay.

A secondary analysis also examined differences between groups pre- and post-COVID-19 to assess the impact of COVID-19 on IVIg prescribing practices. The cases in the pre-COVID-19 group were defined as anyone receiving immunoglobulin therapy before 31st December 2019 when the first confirmed cases were reported by the World Health Organization (WHO). The first confirmed case of coronavirus entered the UK on 23 January 2020. No cases of IVIg were administered between these dates.

### 2.3. Statistical Analysis

Descriptive statistics for participant characteristics were reported using mean +/− standard deviation, and where appropriate percentages. Unpaired *t*-tests were used to compare the means of the responders versus non-responders and pre- and post-COVID-19 groups for statistical significance. Categorical variables were analysed using a chi-squared test and a *p*-value of <0.05 was taken as significant.

## 3. Results

Of the 978 patients diagnosed with CDI [hospital- (617) and community-onset CDI (361)] over the 5-year study period, 20 patients were treated with IVIg. The Supplementary Table S1 summarizes the patients’ characteristics. Table 1 shows the baseline characteristics and treatment outcomes with IVIg. Of the 20 patients that received IVIg, 11 (55%) responded to treatment based on the resolution of symptoms. The mean total dose of IVIg administered across the whole cohort was 33.2 g (SD 15.5). The mean number of days to disease resolution in IVIg responders was 8.6 (SD 10.7). Notably however, there was a subset of responders who improved particularly quickly; six patients demonstrated disease improvement/resolution within 2 days (two had rCDI and four severe disease). Of note, one patient (case T, Table S1) who presented with severe rCDI (six clinical CDI episodes with hospitalization) failed two faecal microbiota transplantations via nasogastric tube but responded to IVIg. All the IVIg patients were of White British ethnic origin and from their own homes. The average age, BMI, and Charlston Comorbidity Index of the cohort were 65.8 years (24.2), 24.3 (5.9), and 5.9 (2.9), respectively, and no significant differences were observed in these variables between responders and non-responders.

Twelve (60%) had hospital-onset CDI. Sixteen (80%) patients were treated for severe CDI and four (20%) either for rCDI (n = 3) or relapsing CDI (n = 1, patient T in Supplementary Table S1), with no statistically significant differences in response to IVIg between these groups (rCDI and relapse combined) (*p* = 0.37). Prior to admission, 7 (35%) patients had a PPI and 13 (65%) patients had received a recent course of antibiotics, with an average number of medications of 7.4 (4.2). None of these were significant between responders and non-responders (*p* = 0.88, *p* = 0.73, *p* = 0.78, respectively).

Nineteen (95.0%) patients had radiological evidence of CDI, ten of whom had pancolitis, and one patient was found to have a toxic megacolon in an abdominal X-ray; one patient had a normal abdominal X-ray. There was no difference in WCC, albumin, or CRP between groups prior to IVIg administration (*p* = 0.92, *p* = 0.85, *p* = 0.93). The average time to IVIg administration after diagnosis was 6.2 (4.9) days, with no difference between groups (*p* = 0.88).

Surgical intervention was required in three (15%) patients; two of those required a subtotal colectomy secondary to CDI and one had emergency surgery secondary to a retroperitoneal bleed and a subtotal colectomy for CDI. The average length of hospital stay for the whole cohort was 30.4 (15.2) days (*p* = 0.47). Two (10%) patients had a recurrence of CDI: one in the responders’ group and one in the non-responders’ group.

The 30-day mortality was 30% (n = 6). Two of those were in the responders’ group. In the non-responders’ group, three out of four (75%) with 30-day mortality had CDI mentioned on the death certificate as a cause of death.

Ribotyping data were available for fifteen patients (Supplementary Table S1); five patients did not have samples sent for ribotyping, and one did not grow *C. difficile* on the sent sample. The ribotypes detected in the cohort were as follows: one each of ribotype 066, 268, 015, 002, and 023, and two each of 014, 078, 011, 050, and 081.

**Figure 1:**
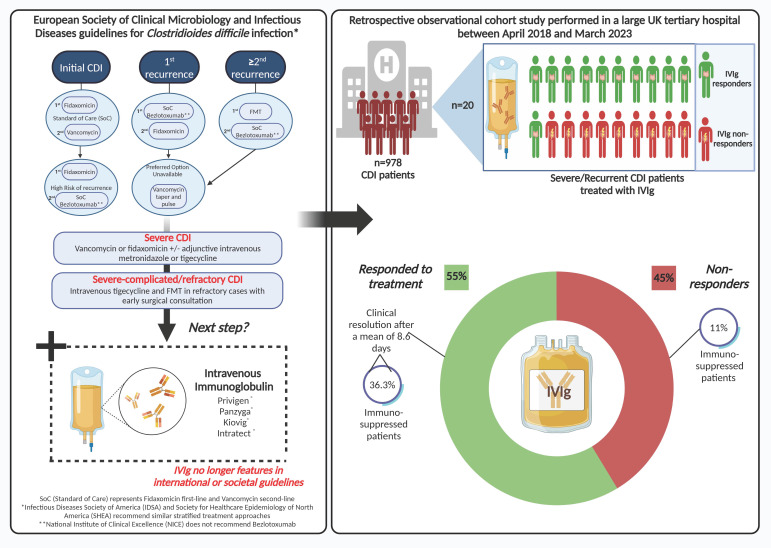
Schematic of main study design and findings.

In the period covered until the outbreak of COVID-19, there were only two patients given IVIg for severe CDI. One (50%) of those responded in the pre-COVID-19 period compared to ten (58%) of those given IVIg post-COVID-19 (*p* = 0.88). Therefore, we were not able to draw any firm conclusions as to the impact of COVID-19 on IVIg response. There were no statistically significant differences in the Charlston Comorbidity Index, age, IVIg doses, time taken to request IVIg, or the time to administration between responders and non-responders (Table 1). A schematic diagram of the study design and results is shown in Figure 1.

Within our single centre, we demonstrated a clinical response to IVIg therapy in more than 50% of patients with refractory CDI. Within this cohort, we found no statistically significant predictors of disease resolution post IVIg administration from date of diagnosis, based on either demographic or clinical features. Furthermore, we detected no statistically significant differences between the groups in any of the secondary outcomes, surgical intervention, length of hospital stay, mortality, or rate of recurrence of infection.

## 4. Discussion

The present study is unique in that it is the first to report on the use of IVIg for severe or rCDI prior to, during, and following the COVID-19 pandemic. We report an overall response rate to IVIg of 55% amongst patients hospitalised with mainly severe (75%) or rCDI. Notably, four (36%) of those who responded were immunosuppressed compared with just one (11%) of the non-responders. Six patients responded rapidly within two days of treatment. Of these six rapid responders, three (27%) were immunosuppressed. In line with similar-sized case series, Abougergi et al. reported that six patients with severe CDI also responded promptly to medical treatment and nine of twenty-one patients survived their illness with colitis resolution [27]. Although the cohort is not sufficiently large enough to draw statistically significant conclusions, there is a possible role of baseline immunosuppression as a factor that may influence treatment response and thus patient selection for IVIg. We are not aware of any previous reports investigating the interaction between patient immunosuppression and IVIg efficacy in the context of CDI. Although speculative, baseline immunosuppression that is associated with a more muted immune response may create a more conducive setting for low-dose IVIg replacement therapy, which can still help dampen the inflammatory process associated with *C. difficile* colitis. In patients with severe CDI who are not immunocompromised and reportedly display increased local and systemic levels of proinflammatory cytokines [30,31,32,33], we hypothesize that an unchecked or exaggerated innate and adaptive immune response characterised by hyperinflammation and cytokine storm may respond to high-dose IVIg, which is used to treat inflammatory and autoimmune conditions; here, the latter higher dosing strategy takes an active part by modulating the immune functions with additional anti-inflammatory activity [34].

In our study, patients experienced clinical resolution after a mean of 8.6 days (SD 10.7) after receiving IVIg, and three patients (15%) required surgical intervention compared to that reported by Abougergi of 10 days and two patients (10%), respectively. In contrast to the latter study where 12 patients (57%) died during the index hospital admission, our findings indicate a lower 30-day mortality of 30% (six patients). In terms of serious adverse events, one patient developed a pulmonary embolism whilst on prophylactic enoxaparin which was detected during CT pulmonary angiography 4 days after the initial IVIg infusion, whereas Abougergi reported one case of pulmonary oedema [27]. In another similar-sized retrospective analysis, Juang et al. evaluated the clinical efficacy of IVIg in 18 patients with severe CDI who were matched to similar controls who did not receive treatment, demonstrating no significant differences between groups [35].

Ten distinct *C. difficile* ribotypes were detected in this retrospective study and these were one each of ribotype 066, 268, 015, 002, and 023, and two each of 014, 078, 011, 050, and 081. Ribotypes such as RT078 seen in the present study, along with RT018, RT027, RT056, RT176, and RT244, have all been reported to be associated with complicated disease outcomes, recurrences, and increased severity [36]. Herbert et al. identified the 10 most common ribotypes in their two-year surveillance period to be RT002, RT015, RT005, RT014, RT020, RT078, RT220, RT108, RT206, and RT023 in descending order, where hypervirulent RT027 was isolated in only five patients (0.7%). RT014 has previously been reported to be associated with increased mortality [37]. An exploratory study which also evaluated *C. difficile* polymerase chain reaction ribotypes and infection outcomes reported a similar level of severity of RT014 to that of RT027 in terms of resolution of diarrhoea and length of hospital stay, which may explain the long hospitalization experienced by patients B and D who had RT014 [38].

In line with a prior case report by Coffman et al., we also observed IVIg treatment success in an older male adult with severe refractory CDI who failed standard therapy including FMT and fidaxomicin [20]. However, in our case, a single IVIg infusion of Kiovig^®^ prevented further recurrent infections for the duration of assessment of 10 months post-infusion (time of writing), whereas in the published case report, IVIg was given over 3 days with high-dose Gamunex-C^®^ 10% (1 g/kg) prescribed on the first day of treatment, with the patient successfully responding to treatment three days post-infusion. Coffman’s case report in conjunction with our own single case observation underscores that not all patients with rCDI respond to FMT and that IVIg still should be considered among the therapeutic arsenals.

Whilst we designed our 5-year retrospective study to span the period before, during and after the COVID-19 pandemic, we only identified two severe CDI cases that received IVIg for CDI prior to the pandemic. Although we could not detect any meaningful differences in patient characteristics and response rates in the pre- and post-COVID-19 periods, our analyses were severely limited by the small sample size in the pre-COVID-19 period. Nonetheless, the post-COVID-19 IVIg cohort displayed a higher Charlson Comorbidity Index compared to the pre-COVID-19 treatment cohort, which may partly explain why we detected a predilection for prescribing IVIg for mainly severe and more complex CDI in the period after the emergence of COVID-19. The stated threshold for prescribing IVIg had not changed in the post-COVID-19 period.

Our study has several limitations. These include its small sample size, retrospective study design without a matching control group, and lack of ribotyping data for the full case cohort. Baseline immune parameters such as immunoglobulin levels, or T- and B-cell immunophenotypic data, were not available. Due to the lack of biobanked sera, we were also not able to determine baseline or longitudinal IgG anti-*C. difficile* toxin antibody levels or functional neutralizing capabilities. It is important to note that, in the UK, IVIg is a limited-availability blood-product therapeutic resource.

Whilst the limited supply of IVIg makes large randomised controlled studies with sufficient power difficult to conduct, larger case–control prospective observational studies coupled to mechanistic studies should be undertaken. These should focus on assessing the role of IVIg in patients with severe CDI (perhaps with a focus on those that are immunosuppressed) and in patients with rCDI that fail FMT, or where the latter is declined or deemed to be inappropriate. Mechanistically, studies should be undertaken to investigate which immune compartments are being modified with treatment over time, aligned with the known multimodal action of IVIg. Such studies could be expanded to also include multi-analyte protein microarray technology to determine binding antibody responses to multiple *C. difficile* antigens, as previously described by our own group [15,39,40]. Microarray technology may help select and optimise the most clinically useful therapies for CDI in a patient-specific manner.

Parallel studies should evaluate the impact of IVIg on the functional capabilities of *C. difficile* toxin-specific antibodies in sera in relation to toxin neutralisation capacity, as mediated by the fragment antigen binding (Fab) region and Fc-dependent antibody effector functions, the latter of which is more complicated than assays that measure antibody binding and toxin neutralisation and remains an unmet area of investigation. Since antibody glycoforms are shaped by infection and vaccination, and modulate Fc-dependent effector function, therapeutic IgG glycosylation status should also be investigated in the context of IVIg and emerging antibody-based therapeutics for CDI [41]. Changes in galactosylation, fucosylation, and sialylation are now well-established factors which drive differential IgG function, ranging from inhibitory/anti-inflammatory to activating complement and promoting antibody-dependent cellular cytotoxicity [42]. Finally, in light of recent antibody-based therapy research which has been stimulated by SARS-CoV-2 and which has been met with some success [43,44,45,46,47], consideration should also be given to the development and testing of IgM-enriched IVIg preparations or the utilisation of hyperimmune IVIg to provide standardised and controlled antibody content from patients that have recovered from CDI who have anti-toxin neutralising antibodies in their plasma. It would also be of interest to investigate the role of combinatorial therapy to enhance the capability of IVIg.

In conclusion, our present findings are consistent with IVIg showing a beneficial effect for some patients with CDI, particularly patients with severe CDI and patients that may be immunosuppressed. We argue that larger case–control studies should be undertaken in order to determine the merits of placing IVIg back into CDI treatment guidelines as an adjunctive treatment, particularly given the current lack of a vaccine on the market to prevent initial or rCDI and the limited availability of bezlotoxumab.

## Figures and Tables

**Table 1:** Table of baseline characteristics and outcomes of hospitalized patients with refractory CDI initiating IVIg.

	Whole Cohort (n = 20)	IVIg Responders (n = 11)	*p* Value
Age (years), mean (SD)	65.8 (24.2)	68.9 (20.0)	0.55
BMI (kg/m^2^), mean (SD)	24.3 (5.9)	25.4 (5.1)	0.39
Female gender, n (%)	11 (55.0%)	5 (45.4%)	0.65
Ethnicity: White British, n (%)	20 (100%)	11 (100%)	
Own home, n (%)	20 (100%)	11 (100%)	
Indication: recurrent or relapsing, n (%)	4 (20.0%)	3 (27.3%)	0.37
Indication: Severe, n (%)	16 (80.0%)	8 (72.7%)	0.37
Total IVIg dose (g), mean (SD)	33.2 (15.5)	33.2 (15.5)	0.63
Days to disease resolution, mean (SD)		8.6 (10.7)	
Charlson Comorbidity Index, mean (SD)	5.9 (2.9)	6.3 (3.2)	0.52
Immunosuppression, n (%)	5 (25.0%)	4 (36.3%)	0.15
Number of other drugs on admission, mean (SD)	7.4 (4.2)	7.1 (4.6)	0.78
PPI on admission, n (%)	7 (35.0%)	4 (20.0%)	0.88
Hospital-onset CDI, n (%)	12 (60.0%)	7 (63.6%)	0.73
Community-onset CDI, n (%)	8 (40.0%)	4 (36.4%)	0.71
Recent antibiotics, n (%)	13 (65.0%)	8 (72.7%)	0.73
Peak WCC (×10^9^/L), mean (SD)	27.8 (15.8)	27.4 (15.4)	0.92
Peak CRP (mg/L), mean (SD)	267.8 (101.5)	265.8 (89.8)	0.93
Lowest albumin (g/L), mean (SD)	18.2 (4.0)	18.4 (3.0)	0.85
Radiological evidence of CDI, n (%)			
Toxic megacolon	1 (5.0%)		
Pancolitis	12 (60.0%)		
Localized colitis	5 (25.0%)		
Chronic colitis	1 (5.0%)		
Normal	1 (5.0%)		
Days from diagnosis of CDI to IVIg request, mean (SD)	5.5 (3.3)	6.36 (4.0)	0.19
Days from onset of symptoms to IVIg administration, mean (SD)	10.3 (5.1)	9.9 (5.5)	0.72
Days from diagnosis of CDI to IVIg administration, mean (SD)	6.2 (4.9)	6.36 (4.10)	0.88
Second infusion required, n (%)	6 (30.0%)		
Surgical intervention, n (%)	3 (15%)		
Length of hospital stay, mean (SD)	30.4 (15.2)	30.5 (14.3)	0.47
Recurrence following IVIg, n (%)	2 (10%)	1 (9.0%)	0.89
30-day mortality, n (%)	6 (30%)		
Mortality attributable to CDI (if 30-day mortality), n (%)	3 (50%)		

Abbreviations: IVIg, intravenous immunoglobulin; BMI, body mass index; PPI, proton pump inhibitor; CDI, *C. difficile* infection; WCC, white cell count; CRP, c-reactive protein; g, grams; SD, standard deviation.

## Data Availability

The data that support the findings of this study are available from the corresponding author upon reasonable request. The data are not publicly available as this study was registered as a service evaluation project and thus permissions would need to be requested from the Clinical Trust to share data sets.

## Supplementary Materials

The following supporting information can be downloaded at: https://www.mdpi.com/article/10.3390/antib13020026/s1, Supplementary Table S1, The individual patient characteristics and presentations of CDI cases treated with IVIg.
